# The new system for classification of periodontal and peri‐implant disease: A questionnaire study of implementation by Swedish dental hygienists

**DOI:** 10.1111/idh.12816

**Published:** 2024-05-09

**Authors:** Sebastian Malmqvist, Patrik Strandberg, Ida Victorin, Emelie Boberg, Annsofi Johannsen

**Affiliations:** ^1^ Division of Oral Diseases, Department of Dental Medicine Karolinska Institutet Huddinge Sweden; ^2^ Danakliniken Specialisttandvård, Praktikertjänst AB Stockholm Sweden; ^3^ Aqua Dental Malmö Sweden

**Keywords:** classification, dental hygienist, peri‐implant diseases, periodontal disease, public dental health service

## Abstract

**Objective:**

To what extent do dental hygienists (DH) employed by the Public Dental Health Service (PDHS) in Sweden use the new classification system, their knowledge of it and their attitudes towards it.

**Methods:**

A web‐based questionnaire was distributed to DHs in the PDHS in different regions of Sweden. A total of 197 registered DHs responded. The questions covered their knowledge, attitudes and possible barriers to implementation of the new classification system of periodontal and peri‐implant diseases, and a question about their perceived need for a complementary digital tool to facilitate its implementation.

**Results:**

Seventy per cent of the DHs stated that they used the new classification system. Twenty‐nine per cent of the participants were confident in classifying periodontitis under the new system. Furthermore, 36% of the participants considered their knowledge of the new system to be good and 33% to be poor or non‐existent. Several DHs stated that the new system was too time‐consuming, that it caused stress, that their knowledge was inadequate and that they, therefore, considered it too difficult to use. Eighty per cent of the participants were positive to a digital tool as a complement and support to classify periodontitis and peri‐implantitis.

**Conclusion:**

The present study showed that most of the DHs used the new classification system and one‐third considered their knowledge to be good, although it was difficult and time‐consuming. Furthermore, in general, the DHs were positive to a digital tool to facilitate application of the new classification system.

## 1 INTRODUCTION

Periodontitis is a common global health problem with a prevalence, in dentate adults, estimated to be around 62% for any stage of the disease and its severe variants 23.6%.^1^ If untreated, the disease can lead to tooth loss and both psychological and physiological sequelae, which also can lead to deterioration of quality of life.^2^, ^3^

A new system for classifying periodontal and peri‐implant diseases and conditions was developed in 2017 and was introduced in 2018.^2^ It has three components: (i) diagnosis of an individual as a periodontitis case; (ii) identification of the specific form of periodontitis; and (iii) case assignment through the novel process of staging and grading.^4^ ^(2)^ In the new classification, periodontitis is stratified into four stages according to the severity and the complexity of managing the disease (stages I–IV). There are three grades (A–C) of risk of disease progression based on radiographic bone loss, clinical attachment loss and risk factors such as smoking and diabetic status.^4^ In addition, each stage of the disease is classified as localized or generalized.

The new system has been used to estimate the prevalence and severity of periodontitis in Norway.^5^ They found a prevalence of 17.6% when combining stage III and IV periodontitis. In another study, the importance of employing a globalized standard case definition was emphasized.^6^ They showed a quite large discrepancy in prevalence (stage III and IV combined) between the new classification system from 2018 at 54% compared to 16.8% when using the system from 2012 by the American Academy of Periodontology/Centers for Disease Control and Prevention (AAP/CDC).

A new category has been introduced, covering peri‐implant health and diseases such as peri‐implant mucositis and peri‐implantitis.^2^, ^7^ The classification focuses on the characteristics of (i) peri‐implant health, (ii) peri‐implant mucositis, (iii) peri‐implantitis and (iv) soft‐ and hard‐tissue deficiencies.^8^ The new system is intended to provide a uniform approach to periodontal and peri‐implant diseases and also allow for effective communication among healthcare professionals, patients and the authorities.^7^

At patient level, the reported prevalence of peri‐implant mucositis is 46.83% and peri‐implantitis 19.83%.^9^ Major risk factors are inadequate plaque control and supportive care after implant treatment.^10^, ^11^ Untreated or unsuccessfully treated peri‐implantitis also has a negative impact on the patient's psychosocial and functional well‐being.^12^, ^13^

The new classification system, introduced in 2017, differs considerably from the previous system from 1999 and may prove a challenge because it differs from the way in which periodontists/dentists usually formulate their diagnoses.^14^, ^15^ There are often challenges associated with applying new knowledge and introducing a new system, including the awareness level, the technical difficulties, the feasibility of application and the size of the gap between theory and practice.^6^, ^15^

It is, therefore, important to ascertain whether dental hygienists (DHs) have the knowledge and competence necessary to diagnose periodontitis and peri‐implantitis.

The aim of the present study was to investigate to what extent dental hygienists employed by the PDHS in Sweden use the new classification system, their knowledge of it and their attitudes towards it.

## 2 STUDY POPULATION AND METHODOLOGY

This study was approved by the Regional Ethics Committee in Stockholm, Sweden (dnr. 2021‐03518). No personal data were collected, and the participants filled in the questionnaire anonymously. The study was designed as a quantitative, questionnaire study and described in accordance with the Standards for Reporting Results of the Internet E‐Surveys (CHERRIES).^16^

The study was conducted as an empirical quantitative study, using a web‐based questionnaire. Stakeholders and the managers who were responsible for research and development within the PDHS in 21 regions in Sweden were contacted by email. Of these, 15 regions agreed to participate in the study. Four regions did not respond at all, and two regions actively declined to participate in the study. The regions which agreed to participate were further contacted and informed about the study and information about the survey was sent out to the clinics. The managers of the participating regions distributed the digital questionnaire to the registered dental hygienists employed in their region. A total of 903 DHs received the questionnaire and 197 participated giving a response rate of 22%. Primary contact with the different regions was established in the spring of 2021, and after the decision by the regional PDHS to participate, the questionnaire was made available on the intranet or via email, depending on the specific region's established and preferred channel of communication, to the dental hygienists for at least 1 month. After at least 2 weeks, a reminder was sent out to the dental hygienists. All questionnaires were filled anonymously.

### 2.1 The questionnaire

The questionnaire was designed by the authors. Some questions were adapted from an earlier study.^17^ In collaboration with 10 DHs who did not participate in the study, the questions were then discussed and refined with reference to readability and understanding.

The questionnaire consisted of 12 questions, 9 regarded the new classification system and 3 questions regarded the DH background. The background questions concerned, for example, gender, age and number of years as a professional DH. The nine questions about the new classification system (eight multiple‐choice and one open question) referred to which system they use today, their reason for not using the new system, their attitudes, knowledge, how they got information about the new system and from what sources and their experience of the new classification system.

One question elicited the respondents' attitudes towards and views of a complementary support function, such as a digital tool, to facilitate application of the new classification system. The final question was open ended and allowed the respondent to elaborate on the new classification system. At all times during the fulfilment of the questionnaire, the respondents were able to review and change their answers, until submission. All 197 received questionnaires were completed and analysed.

### 2.2 Analysis

The survey responses were automatically coded and thereafter compiled for further processing using Microsoft Excel (Microsoft Corporation, Redmond, WA, USA). The questions were categorized as introductory, intermediate or closing questions respectively. Descriptive statistical methods were used, and the data are presented in percentages and numbers.

## 3 RESULTS

The study participants comprised 197 DHs employed by the PDHS in Sweden (Table 1). The majority were women 96% (*n* = 189). The age range was 22–67 years, with an average age of 45 years. Their experience as DHs ranged from none to 39 years, with an average of 15 years.

**TABLE 1:** Number (*n*) and proportion (%) of potential and actual responders in the partaking regions.

Region	Potential responders (*n*)	Responded (*n*)	Proportion (%)
Blekinge	40	13	33
Dalarna	55	16	29
Gotland	10	4	40
Gävleborg	117	24	21
Halland	44	1	2
Jämtland Härjedalen	27	6	22
Jönköpings län	14	0	0
Kalmar	60	25	42
Norrbotten	73	14	19
Stockholm	143	10	7
Uppsala	93	18	19
Västernorrland	45	14	31
Västmanland	48	9	19
Örebro län	92	11	12
Östergötland	93	30	32
Total	903	197	22

The results are presented in Table 2. Seventy per cent of the DHs stated that they used the new classification system from 2017. Twenty‐four per cent used the classification from 1999, 2% used the periodontal classification from 1989 and 4% used another system. When asked why they did not use the new classification, 26% (*n* = 51) stated that it was because their knowledge of the new system was inadequate, 12% (*n* = 23) were more comfortable with their old system and 11% (*n* = 22) did not know (more than one option could be chosen).

**TABLE 2:** Questions put to the dental hygienists about issues related to the new classification for periodontal and peri‐implant diseases, presented in percentages (%) and numbers (*n*).

Questions, percentages and number	% (*n*)	% (*n*)	% (*n*)	% (*n*)
Which classification system do you use when diagnosing patients with periodontitis?	The new system from 2017: 70 (138)	The system from 1999: 24 (47)	The system from 1989: 2 (4)	Other system: 4 (8)
Reason to not use the new classification system^a^	Didn't have knowledge about the new system: 26 (51)	More comfortable with the old system: 12 (23)	Didn't know: 11 (22)	
Confident in classifying periodontal disease according to the classification system you use today. (Regardless of which system they used)	Very Confident 8 (16)	Confident 39 (77)	Not confident 30 (59)	No answer 23 (45)
Confident in classifying periodontal diseases according to the new classification system	Very confident: 3 (5)	Confident: 26 (51)	Don't know 23 (45)	Not confident 48 (96)
Knowledge about the new classification of periodontal and peri‐implant diseases	Good knowledge 36 (71)	Fair knowledge 31 (61)	Little knowledge 27 (53)	No knowledge 6 (12)
Perception of the information received about the new classification	Good 47 (92)	Neither good nor bad 35 (68)	Bad 18 (37)	
Sources of information about the new system^a^	Workplace 73 (143)	Colleagues 43 (84)	Course in periodontology 26 (51)	
A digital tool to support the classification	Positive attitude 80 (157)	No opinion 18 (35)	Negative attitude 2 (5)	

^a^ It was possible to give more than one alternative.

In response to the question about the DHs' level of confidence in classifying periodontal diseases (regardless of classification system), 47% stated that they were confident, while 30% stated that they were not confident and 23% did not respond. Furthermore, in response to the question of how confident the DHs were in using the 2017 system, 29% of the DHs reported that they felt confident, 23% did not know and 48% were not confident.

With respect to knowledge of the new classification, 36% of the DHs stated their knowledge to be good, 31% fair, and 27% and 6%, respectively, reported that they had little or no knowledge of the system (Table 2).

Nearly half (47%) of the DHs perceived that the information they received about the new system was thorough and easy to understand. Most of the DHs (73%) had received information about the new system from their workplace, 43% from colleagues and 26% from a course in periodontology. Other sources of information were a Dental Congress, a dental hygienist journal, dental hygienist undergraduate education or through the internet or social media. Through the open‐ended question, most DHs stated that they considered the new system to be difficult to use, time‐consuming and causing stress. These drawbacks hindered the classification system from being incorporated into the DHs' daily work routine.

Examples of quotes from the dental hygienists:

‘I don't have enough knowledge’.

‘The clinic where I work does not use the new system’.

‘I have tried to use the new system but due to lack of time it's easier with the old one’.

‘It takes a long time to use, it is unnecessary and complicated, I see no benefit of using the system because different therapists assess the same situations differently’.

‘The system is too complicated with too many things to think about’.

‘Due to stress in my daily work, it takes too long to use the new system’.

Most of the participants (80%) believed that a digital tool would be beneficial as a complementary support to application of the periodontal classification system, whereas 20% had no opinion or were negative to a digital tool (Table 2).

## 4 DISCUSSION

The aim of the present study was to explore the degree to which the new classification system from 2017 was used among DHs in PDHS. Seventy per cent of the DHs stated that they were using the new system. However, only 36% reported that they had a good knowledge of the system, while 29% were confident in applying it to classify periodontal diseases. In a similar study by Hegab and Abdelkawy,^17^ 24% of the participating Egyptian periodontists answered that they had implemented the new classification system in their clinical practice. This study was conducted in 2020, and one can expect that today a much higher proportion of Egyptian periodontists would have adopted the system. Comparison with the present study is, however, difficult because periodontists treat primarily patients with periodontitis and DHs in the Swedish PDHS treat diverse patient groups, including children and young people, and treatment is based on health promotion. Furthermore, both the participants in the present study and those from Hegab and Abdelkawy^17^ perceived the new system to be more complicated and time‐consuming than the previous classification system.

It is of great importance for the DH profession to be able to correctly classify periodontitis as approximately 80%–90% of the DHs’ work with this patient category.^18^ Over the last 40 years, the number of remaining teeth has increased in the ageing population, meaning that there is also an increased need for supportive care for a larger number of teeth affected by periodontitis.^19^

Abrahamian et al.^20^ evaluated the intra‐ and inter‐examiner reliability of diagnosis according to the new system. A total of 174 dentists participated in the study including specialists, post‐graduates and academics. The results showed a high level of validity and reliability among postgraduate students and specialists applying the new classification. In the present study, we did not investigate the examiner's reliability, which would have been of interest, because DHs in Sweden both diagnose and undertake non‐surgical treatment on patients with periodontal disease. It would be of interest to undertake a further study, similar to Abrahamian et al.,^20^ but including dental hygienists and dentists.

Marini et al.^21^ found that education and practical skills play an important role in disease classification. Their study evaluated consistency and accuracy of the periodontitis staging and grading classification system among 30 participants (10 periodontists, 10 general dentists and 10 dental students). The results showed that classification by general dentists was generally poorer than by dental students and periodontists. While no such comparison was undertaken in the present study, the importance of practical skills should not be underestimated.

Other important issues warranting investigation are whether the new system has been integrated into the dental and dental hygiene undergraduate programmes and whether there are any obstacles to integrating the system. Aboalsaud et al.^22^ looked at 140 dental hygiene undergraduate programmes in the United States, Canada and Australia, which had integrated the new staging and grading system in their curriculum, both in theoretical and clinical courses (99% and 94%, respectively). The main barriers to implementation were lack of faculty support, lack of information and continuing education courses covering the new staging and grading system and failure to allow sufficient time to make the adjustments necessary for implementation. In the dental hygiene undergraduate programme in Sweden, the new classification is included in the curriculum. However, to our knowledge, there is to date no study investigating and evaluating the students' knowledge of the new system. Within the framework of DH's competence, their work must be evidence based.^23^ Cobban and Profetto‐McGrath highlighted the need for greater understanding of the interactions among the individual, the research findings and the practice context.^24^

The structure of the new classification system promotes individualized, patient‐centred care, which is important for the provision of optimal oral health care. The new system also facilitates interprofessional collaboration by standardizing case definitions and thus enabling easier clearer communication around individual patient cases.^7^ Both diabetes and smoking have been included as risk factors in the new classification system. It has also been confirmed that in patients with type 2 diabetes, non‐surgical treatment has an impact on metabolic control and reduction of systemic inflammation.^25^ The new classification system does, however, need further development: for example, there is to date no provision for staging of peri‐implantitis.

Altogether, these findings indicate that successful adoption of the new classification system requires considerable additional training. In this context, training and implementation seem to be critical: imprecision and misclassification might limit the potential health gains achievable by implementation of the new classification.^26^

Most (80%) DHs were positive about a digital tool and believed that it could both supplement and support work with the classification system. Digital technology offers tools that could facilitate and improve decision‐making and by extension, optimize the patient's care.^27^, ^28^ Under the new classification system, the individual clinician's input and reasoning are still important^29^ and should be emphasized when developing a digital tool for assisting in classification.

The present study has some limitations with respect to recruitment of participants: of 21 regions in Sweden, only 15 agreed to participate. The survey was distributed to stakeholders/managers of each region, who in turn forwarded the survey to the clinical managers of the respective clinic, who then distributed it to the DHs. This involvement of numerous people in the distribution process could have had an effect on the number of responses: recruitment resulted in a 22% response rate (197 of 903 available DHs) and it is, therefore, difficult to draw any general conclusions. A dropout analysis would have been desirable, unfortunately, however, we were unable to reach the non‐responders. It is likely that one of the reasons for not participating in the study could be that they (both the clinics and DHs) did not use the new classification system or had no knowledge about it. However, the sample in the present study showed similar distribution between women and men with 96% of responders being women, compared to 95% of women for DHs overall in Sweden according to data from the National Board of Health and Welfare.^30^ Another drawback is that the study was undertaken during the COVID‐19 pandemic, and this may have restricted participation rates and the representativeness of the results. Furthermore, DHs working in the private sector were not included, which is a limitation. We chose the PDHS since we were expecting a more homogenous group of DHs and, thus more comparable results. To reach all private DHs is also difficult, however, we plan to include them in a future study.

Despite the limitations, this study highlights an important issue. Knowledge of the new classification system is essential, as is an understanding of how to apply it, to ensure that treatment of the disease is based on the evidence. Further follow‐up studies are warranted to investigate the implementation of the new classification system by both dental hygienists and dentists and to investigate further the barriers encountered and to what extent they affect the implementation of the new classification system in daily clinical practice.

## 5 CONCLUSIONS

The present study showed that most of the DHs used the new classification system and one‐third considered their knowledge to be good, although it was difficult and time‐consuming. Furthermore, in general, the DHs were positive to a digital tool to facilitate application of the new classification system.

## 6 CLINICAL RELEVANCE

### 6.1 Scientific Rationale for the Study

The scientific rationale for this study was to determine whether the new classification system is being applied by dental hygienists in clinical practice and their opinion of it.

### 6.2 Principal Findings

Approximately half of the participants used the new classification system daily, but most of them did not feel very confident. The majority stated that the new system was time‐consuming and difficult to use, which limited the implementation.

### 6.3 Practical Implications

It is of great importance to explore and acknowledge the experiences of DHs and their opinions of the new classification system and to explore any potential barriers to its application in their daily work.

## AUTHOR CONTRIBUTIONS

Sebastian Malmqvist – Concept/design, assisting in data collection, data analysis, drafting, critical revision of article and approval of the manuscript. Patrik Strandberg – Concept/design, assisting in data collection, data analysis, drafting, critical revision of article and approval of the manuscript. Ida Victorin – Concept/design, assisting in data collection, data analysis, drafting, critical revision of article and approval of the manuscript. Emelie Boberg – Critical revision of article and approval of the manuscript. Annsofi Johannsen – Concept/design, drafting, critical revision of article, approval of the manuscript, securing funding for the study.

## FUNDING INFORMATION

This study was supported by the Karolinska Institutet.

## CONFLICT OF INTEREST STATEMENT

The authors declare that they have no conflict of interest.

## Data Availability

The data that support the findings of this study are available from the corresponding author upon reasonable request.
